# Patients’ goals when initiating long-acting injectable buprenorphine treatment for opioid use disorder: findings from a longitudinal qualitative study

**DOI:** 10.1186/s13011-023-00551-0

**Published:** 2023-06-22

**Authors:** Joanne Neale, Stephen Parkin, John Strang

**Affiliations:** 1 National Addiction Centre, Institute of Psychiatry, Psychology & Neuroscience, King’s College London, London, SE5 8BB UK; 2 Centre for Social Research in Health, University of New South Wales, Sydney, NSW 2052 Australia; 3 South London & Maudsley (SLaM) NHS Foundation Trust, London, SE5 8AZ UK

**Keywords:** Abstinence, Long-acting Injectable Buprenorphine, Longitudinal, Opioid use disorder, Person-Centred Care, Recovery, Substance use, Qualitative

## Abstract

**Background:**

Long-acting injectable buprenorphine (LAIB) is a new treatment for opioid use disorder that has been introduced against an international policy backdrop of recovery and person-centred care. This paper explores the goals that people want to achieve from LAIB to identify potential implications for policy and practice.

**Methods:**

Data derive from longitudinal qualitative interviews conducted with 26 people (18 male; 8 female) initiating LAIB in England and Wales, UK (June 2021-March 2022). Participants were interviewed up to five times by telephone over six months (107 interviews in total). Transcribed interview data relating to each participant’s treatment goals were coded, summarised in Excel, and then analysed via a process of Iterative Categorization.

**Results:**

Participants often articulated a desire to be abstinent without defining exactly what they meant by this. Most intended to reduce their dosage of LAIB but did not want to rush. Although participants seldom used the term ‘recovery’, almost all identified objectives consistent with current definitions of this concept. Participants articulated broadly consistent goals over time, although some extended the timeframes for achieving treatment-related goals at later interviews. At their last interview, most participants remained on LAIB, and there were reports that the medication was enabling positive outcomes. Despite this, participants were aware of the complex personal, service-level, and situational factors that hindered their treatment progress, understood the additional support they needed to achieve their goals, and voiced frustrations when services failed them.

**Conclusions:**

There is a need for wider debate regarding the goals people initiating LAIB are seeking and the diverse range of positive treatment outcomes LAIB could potentially generate. Those providing LAIB should offer regular on-going contact and other forms of non-medical support so that patients have the best opportunity to succeed. Policies relating to recovery and person-centred care have previously been criticised for responsibilising patients and service users to take better care of themselves and to change their own lives. In contrast, our findings suggest that these policies may, in fact, be empowering people to expect a greater range of support as part of the package of care they receive from service providers.

## Background

In recent years, treatment options for opioid use disorder have expanded with the emergence of new types of medication, including long-acting formulations that bypass the need for daily dosing [1–3]. Since 2017, three long-acting injectable buprenorphine (LAIB) medications (Sublocade, weekly Buvidal, and monthly Buvidal) have come to market, with studies indicating that these new treatments can reduce opioid withdrawal symptoms and the desire to use opioids, increase treatment adherence and abstinence from non-prescribed opioids, and help patients regain control over their daily lives [4–7]. Despite this, some patients experience negative side effects which cause them to discontinue the treatment [6, 8]. Meanwhile, other people express anxiety regarding initiating LAIB because they are concerned about the medication’s effectiveness, lack of social contact when service attendance reduces, and stopping treatment once started [9–11]. To-date, however, there has been a lack of research exploring what people initiating LAIB want to achieve from the treatment.

Over the last three decades, the drug policy landscape has evolved, and two important developments have been the rise of the recovery agenda and an increasing focus on personalisation. The term ‘recovery’ has its origins in self-help discourses [12] and 12-step fellowships [13, 14], but its meaning continues to evolve [15–18]. During the 1990s, a new recovery discourse emerged in the United States and subsequently spread to other countries, including the United Kingdom (UK) [19, 20]. In the UK, stakeholders argued that this new recovery was characterised by ‘voluntary sustained control over substance use which maximises health and well-being, and participation in the rights, roles and responsibilities of society’ [21]. Responding to this, critical policy scholars, largely based in Australia, warned that framing recovery in this way can erase the political, economic, legal, and cultural relations that shape people’s opportunities. Equally, it can responsibilise people who use substances to monitor, control and change aspects of their lives over which they may have little control [19, 20, 22, 23].

Embedded within recovery discourse is the concept of individual choice, which is also a core component of personalisation and person-centred care. Personalisation has been central to international social care policy and practice for several years [24–27] and is now playing an increasing role in the provision of medical treatment in the UK [28]. Providing person-centred care and support can take many forms, including enabling people who use drugs to have choice and influence over their treatment goals and the way care is planned and delivered to achieve those goals. People who use services and patients are placed at the centre of service provision to ensure that the support they receive is individually tailored to meet their needs and aspirations [29–31]. This is then actioned by working with people holistically and flexibly and involving them in treatment decision-making [32].

Like the concept of recovery, however, personalisation has been criticised for promoting neo-liberal notions of individualism, expecting those who need services to take more care of themselves, and failing to recognise that structural factors (such as limited budgets, bureaucracy and entrenched organisational practices) can impede personalised ways of working [26, 31–33]. In relation to medications for opioid use disorder, for example, regulatory frameworks, commissioning practices, cost, and individual service provider preferences can all restrict what treatments are offered to people. Furthermore, a patient’s potential to choose a medication best suited to them may be undermined if they do not have access to trustworthy and balanced information about all the available treatment options, or if they are disaffected with their current treatment and so eager to try any alternative [34].

This paper aims to address a gap in the existing literature by exploring what goals patients initiating LAIB for opioid use disorder seek to achieve, including whether their goals change over the first six months of treatment, and what factors participants identify as hindering or enabling their ability to attain their goals. Findings are then discussed with reference to the concepts of recovery and person-centred care to identify potential implications for policy and practice.

## Methods

The data presented are part of an on-going longitudinal qualitative study exploring patients’ views and experiences of receiving LAIB [34, 35]. Participants were recruited between June 2021 and March 2022 from six community drug treatment services located in England and Wales. The study received ethical approval from King’s College London Psychiatry, Nursing and Midwifery Research Ethics Subcommittee (reference: MOD-20/21-15027) with additional approvals secured from the participating treatment services.

At the time of data collection, LAIB was a relatively new treatment in the UK, availability was limited, and weekly and monthly Buvidal were the only products licensed. Although Buvidal is approved for the treatment of opioid use disorder in people aged 16 years or over, there are currently no reliable published UK figures on who is receiving it. The medication is not restricted for use in any specific patient sub-group, but it is generally deemed less suitable for people who prefer methadone, are on high doses of methadone, or have significant liver disease, and specific support considerations may be appropriate if prescribing to patients with anxiety or past trauma. Since there is no maximum recommended duration of treatment and there are no national treatment guidelines, patients can theoretically remain on Buvidal indefinitely and prescribing services have developed their own local treatment protocols (Camurus, personal communication, June 2, 2023).

Clinicians in the six participating services provided any patients who were about to initiate Buvidal with the study information sheet and consent form and gave them basic verbal information about the research. Forty-eight patients expressed interest and agreed that their contact details could be passed to the research team. The researchers established telephone contact with 37 of these 48 individuals and 26 agreed to join the study. These included 18 males and 8 females, ages 30–62 years. Most (n = 20) were White British, English, or Welsh. Twenty-four were being treated for heroin use and two were being treated for other opioids. Their heroin use ranged from 3 to 35 years (mean 14.5 years) and fourteen had ever injected. Just under a third (n = 9) were in a relationship; most (n = 19) were neither working nor studying; and many reported physical (n = 10) and/or mental (n = 13) health problems (see Table 1).

**Table 1:** Participant characteristics (self-reported)

Characteristic	N = 26
**Sex**	
Male Female	18 8
**Age (years)**	
Mean (range)	42 (30–62)
**Ethnicity**	
White British, English or Welsh White Other African Caribbean Asian-Indian Black British Caribbean Mixed Heritage	20 2 1 1 1 1
**Type of opioid being treated**	
Heroin Codeine & Tramadol Methadone	24 1 1
**Length of heroin use (years)** ^†^	
Mean (range)	14.5 (3–35)
**Ever injected**	
Yes	14
**Relationship status**	
In a relationship Separated Single Divorced Widowed	9 8 7 1 1
**Current employment status**	
Not working/ benefits Working full-time Working part-time Student	19 2 2 3
**Current physical health problem**	10
**Current mental health problem**	13

^†^24 participants only

Fieldwork involved repeated semi-structured telephone interviews with the 26 participants. For this paper, data from the interviews conducted at the first five study timepoints (T) are analysed: within 72 h of the first buprenorphine injection (T1); after one week (T2); after one month (T3); after 3 months (T4); and after 6 months (T5). These timepoints were chosen for a combination of treatment-related and methodological reasons. Specifically, we wanted to interview people (i) around the time of their injections (particularly their initial injections) to optimise recall of their experiences and (ii) at regular intervals to minimise attrition from the study. Based on previous research exploring patients’ views of receiving LAIB [10], we anticipated that participants would likely begin with a weekly Buvidal injection (to assess whether the treatment suited them) and then progress to a monthly formulation.

The number of interviews conducted at each timepoint ranged from 26 at T1 to 17 at T5 (24 interviews were conducted at T2 and 20 interviews were conducted at both T3 and T4). Thus, the total number of interviews completed was 107 (26 + 24 + 20 + 20 + 17) out of a possible 130 (15 individuals completed all five interviews). Participants re-consented to participate in the research prior to each interview and were invited to choose either £20 cash or a £20 shopping voucher as thanks on completion of each interview.

The T1 interviews were guided by a topic guide that covered participants’ background; substance use; prior treatment experiences; decision to have Buvidal; experiences of having the first injection; feelings since having the first injection; satisfaction with treatment so far; and future treatment expectations. The topic guides used in subsequent interviews (T2-T5) followed a similar format to facilitate comparison over time and included: life and treatment-related changes since the last interview; experiences of treatment since the last interview; decision-making in relation to having another injection; and future treatment expectations. All interviews were audio-recorded and transcribed verbatim by a professional transcription service. The transcribed interview data were then entered into the software programme MAXQDA version 18 [36] for line-by-line coding, with separate, but similar, coding frames used for each wave of interviewing.

The topic guide sections that focused on participants’ future treatment expectations included questions relating to what participants hoped and expected to achieve from Buvidal and what they hoped and expected their lives might be like after a few months. Participants’ responses to these questions were indexed to codes within each coding frame and these data were exported to a single Word document (labelled ‘Goal Codings’) for the current analyses. This Word document was used to populate an Excel file that had a row (n = 26) for each participant and a column (n = 5) for each timepoint (26 × 5 cells). Each participant’s treatment goals (broadly conceived to include any plans, wishes, hopes or expectations) were next summarised into the relevant cell. The original interview transcriptions were also reviewed to ensure that the Excel file was completed as comprehensively as possible.

Following the above, each participant’s data (across all timepoints available) were summarised in a sixth column in the Excel file (one additional cell per participant) and data from this sixth column were analysed through a process of Iterative Categorization [37, 38]. This involved transferring all the data in the sixth column of the Excel file to a new Word document (labelled ‘Goals Analyses’). All the data in the ‘Goals Analyses’ Word document were then converted into bullet points and each bullet point was labelled with the relevant participant’s study number (01–26) and the timepoint at which the data were generated (T1-T5). The labelled bullet points were next reviewed, organised into themes, and grouped under main and sub-headings. In this way, the Iterative Categorization process enabled us to see where participants agreed or disagreed on issues, review whether and how their viewpoints changed over time, and construct a narrative of the findings.

To help manage the large volume of data, the analytic process had so far been reductive (moving from transcriptions to coded data to summarised data to themes and headings). However, the next stage involved moving back through the headings, themes, summarised data, coded data, and transcriptions to check the findings and supplement the ‘Goals Analyses’ Word document with more detail and quotations. At this point, some of the headings were relabelled and reorganised to ensure optimal fit with the data.

To minimise the likelihood of bias and increase analytic rigour, two team members (SP and JN) worked closely together at all stages of data generation, coding, and analyses. Thus, SP conducted all the interviews and coded all the transcriptions, whilst JN listened to all audio recordings of the interviews and reviewed the coded data. Both SP and JN then contributed to the completion of the Excel file. Subsequently, JN led on organising the data into themes ordered under main and sub-headings and SP critically reviewed the findings. Lastly, all three team members discussed and agreed the structure of the manuscript.

## Results

Participants’ goals could be described under three main headings (‘Goal type’, ‘Goals over time’, and ‘Goal mediators and moderators’) with eight sub-headings (‘Substance use goals’, ‘LAIB treatment goals’, ‘Life goals’, ‘Goal consistency’, ‘Evolving treatment goals’, ‘Disrupted treatment goals’, ‘Barriers to achieving goals’, and ‘Enablers to achieving goals’). Findings are presented under these main and sub-headings below, and also summarised in Table 2.

**Table 2:** Summary of participants’ goals

**Goal type**
***Substance use goals***: Many participants wanted to be ‘drug free’ or ‘abstinent’ but did not clearly define these terms. Several were uncertain about whether they would continue to use non-opioid drugs.
***Long-acting injectable buprenorphine (LAIB) treatment goals***: Most participants wanted to reduce their LAIB and come off ‘at some point’ in the future. However, they often felt that there was no rush. Only two participants wanted to come off LAIB quickly. Some did not want to think about the future of their LAIB treatment.
***Life goals***: Almost all participants wanted to achieve life goals relating to relationships, education, employment, housing, health, travel, hobbies, new routines etc. Few explicitly used the term ‘recovery’.
**Goals over time**
***Goal consistency***: Participants generally reported consistent substance use, treatment, and broader life goals over time.
***Evolving treatment goals***: Participants’ treatment goals sometimes evolved over time, moving from modest initial goals to more firm and ambitious treatment reduction plans at later interviews. Several participants, conversely, extended the time they wanted to remain on LAIB as the study progressed.
***Disrupted treatment goals***: At their last completed interview, a small number of participants had stopped receiving LAIB (so disrupting their treatment goals). One participant had discontinued LAIB as she had not felt comfortable on the medication and three had been removed from LAIB by their service providers for not adhering to treatment protocols.
**Goal mediators and moderators**
***Barriers to achieving goals***: Barriers to making progress towards treatment goals included personal poor health, lack of support from treatment services, and situational factors (e.g., homelessness and unstable housing, insufficient informal support, difficulty avoiding others who used drugs, lack of daily routines and meaningful activity, the closure of services because of COVID-19, and feeling uncertain about the future).
***Enablers to achieving goals***: Factors that helped participants progress towards their goals included having supportive partners and family members, interests or activities that provided routine and structure, access to mutual aid meetings, and paid employment.

### Goal type

#### Substance use goals

At all timepoints (T1-T5), many participants stated that they wanted to be ‘drug-free’, ‘clean’, ‘abstinent’, or ‘not using at all’. Nonetheless, it was frequently difficult to ascertain whether participants meant that they wanted to be abstinent only from heroin or also from other substances, including alcohol, tobacco, and medications prescribed for substance use disorder. Some, such as Participant 01, thought that they might want to be abstinent from both illicit substances and prescribed medications. Others, such as Participant 04, said that they hoped to be maintained on medication for opioid use disorder for the foreseeable future:

“Maintenance for now… I’m thinking a couple of years if I could [stay] on the Buvidal, and then look at lowering it and hopefully one day being total substance free. That’s me objective.” (Participant 04, male, T1)

In addition, several participants expressed uncertainty about whether they would continue to use non-opioid drugs. This included a few who gave no indication of wanting to stop using crack-cocaine, alcohol or cannabis and others who reported that they were worried about their use of other drugs but would address this after they had stopped using heroin:

“Like baby steps, first adjusting to life heroin-free. Ultimately, down the line, I will address my crack issue… I would like to address it.” (Participant 14, male, T2)

At T1, only one participant volunteered that he wanted to keep using heroin. This participant stated that, after 72 h, he already felt ‘trapped’ by LAIB. Nonetheless, he rationalised that continuing to use heroin was not an option for him and he had to abstain from the drug as it was interfering with his relationships, studying and work. This participant was unfortunately lost to follow-up after T1:

“I’m not getting clean because I want to get clean. I’m getting clean because I have to… I’ve got a lot of things going on in my life… I’ve got responsibilities.” (Participant 16, male, T1)

#### Long-acting injectable buprenorphine treatment goals

When asked about LAIB specifically, only a few participants indicated that they wanted to be maintained on the treatment indefinitely. Most stated that their aim was to reduce their dosage and come off at some point in the future; nonetheless, the period they gave for reduction and stopping was variable. Whilst some had not yet thought about when they wanted their LAIB treatment to end, others referred loosely to a period of months and, occasionally, one or two years. Frequently, participants emphasised that there was no urgency, and they did not want to rush:

“I aim to be off it [LAIB] like in two years’ time, so gradually… Over a two-year period, I would like to reduce gradually.” (Participant 26, female, T2)

Sometimes participants added that they did not want to reduce their medication prematurely as this would risk repeating ‘mistakes’ that they had made with other treatments previously. Moreover, they wanted to make this their last ever treatment. Thus, they said that they first preferred to stabilise their dose, address other issues in their lives, and wait for ‘life to improve a bit’. As Participant 08 stated at his T3 interview: “I don’t want to get too far ahead of myself.”

One participant at T1 also explained that he was on a six-month treatment plan that had been decided by his doctor, and he therefore believed that he would have to reduce rapidly. However, he remained on the same dose of LAIB until T5. At T1, only two participants said that they wanted to come off LAIB quickly. By T4, one of these had been taken off the medication because he did not attend for his third injection and the second had been persuaded to wait and reduce more gradually by his doctor:

“She [doctor] said, ‘Look, what you should do is this… then we’ll reduce you down,’ she said, ‘rather than just stopping dead’… And then she got me on board with that.” (Participant 13, male, T4)

Finally, some participants stated that they did not want to think about the future of their LAIB treatment and/or were happy to trust their doctors to make appropriate decisions for them:

“I’m not sure how long the plans are, but knowing [drug service], they’ll keep me on Buvidal for as long as I need to be… Because they do understand like the history of drugs and stuff… Probably wean me off slowly, give me a slower, slower dose, and ask me… ‘Are you comfortable? Are you ready to come down?’” (Participant 17, male, T3)

#### Life goals

Almost all participants between T1 and T5 stated that they wanted to achieve a range of life goals. These included improving relationships with others (family and friends, but especially children); completing education or training; securing paid work or volunteering; moving into stable housing; being more physically active (such as going to the gym); addressing health problems (particularly dental issues and poor mental health); establishing a healthier weight; going on holiday abroad; enjoying old and new hobbies; starting new routines (including cooking); and completing life administration (such as paying bills). At T1, for example, Participant 10 wanted to return to work and have his children visit for the weekends; Participant 14 hoped to spend more time with his daughter and go to the gym; and Participant 23 stated that she wanted to become a mental health support worker. Importantly, however, one participant cautioned that such life changes might be expecting too much:

“All of that wanting to rush back to work, rush back to relationships, and normality. You’re just not ready for it. You’ve just come from the war zone… you’re just not ready to start engaging in normal life.” (Participant 21, male, T4)

Only two participants used the term ‘recovery’ when describing their treatment goals, although a small number of others referred to themselves as ‘being in recovery’. Thus, Participant 04 stated that he would always be ‘a recovering addict’ and Participant 21 spoke of a friend who ‘knows I’m in recovery’. In addition, two participants reported that they would like to become ‘recovery workers’ in the future. None of these participants discussed the concept of recovery in any depth.

### Goals over time

#### Goal consistency

Generally, participants reported consistent substance use, treatment, and broader life goals at each timepoint; that is, they tended to repeat the same or very similar goals over time. Illustrating this, Participant 02 described his desire to be ‘drug-free’ at T1, T2, T4 and T5 (despite being removed from treatment at T3) whilst Participant 08 articulated a preference for receiving a maintenance dose of LAIB at T1, T2, T4 and T5. Participant 15 wanted to travel and reconnect with family at all five interviews and Participant 23 reaffirmed her T1 intention to complete her studies and become a mental health support worker at T3 and T4:

“I’m at college… I do want to become like a support worker, mental health worker, whether it be drug-related or not.” (Participant 23, female, T3)

“I’m hoping to become a mental health support worker… I’ve enrolled onto a couple of short courses… and I’ve also got an appointment with a careers adviser to give me a bit of direction.” (Participant 23, female, T4)

#### Evolving treatment goals

Notwithstanding this general consistency, participants’ treatment goals also sometimes evolved over time. Accordingly, a few participants described quite modest goals initially (e.g., providing a drug-free urine screen or stabilising their buprenorphine dosage), but then advanced to more firm and ambitious plans for reducing by their later interviews. Illustrating this, Participant 19 wanted to give a negative urine screen at T1, talked about reducing his treatment once he was stable at T3, and articulated more concrete plans for reducing and coming off all opioid medication at T5. Not dissimilarly, Participant 20 wanted to continue his medication at T1, aspired to a stable monthly dose with a view to coming off at T2, planned two more monthly injections before reducing and eventually coming off at T3, and was actively planning his reduction to be off his medication at T5:

“He [support worker] has known I’ve wanted to come off this medication. It wasn’t a forever thing… And he knows that I’m sort of ready now. Because I think I’m ready… He’s arranged with the doctor and the nurses to sort of have a plan put in place… I’m not 100% sure what that plan is yet, because my consultation with the doctor’s next week, but I’ve got the gist of it… a reduction this month, and a further reduction next month, and then… holistic sort of therapy after that.” (Participant 20, male, T5)

Conversely, several participants extended the time they wanted to remain on LAIB as the study progressed. For example, at T3, Participant 09 stated that he might start to reduce his LAIB dosage after six months but, at T5, anticipated being on LAIB for a year. Similarly, Participant 15 wanted to cease LAIB within three months at T1, but extended this at each interview and, by T5, wanted to be on LAIB for another five months. Participant 19 recognised his tendency to defer treatment goals as follows:

“I really want to just be properly clean. Hopefully by the end of this year… I think it’s a realistic target, but I did say the same thing last year, so we’ll see.” (Participant 19, male, T3)

#### Disrupted treatment goals

Although most participants were still in treatment at their last completed interview, four had stopped receiving LAIB; a change which had disrupted their treatment goals. Participant 07 had felt uncomfortable during her first month on the medication and had been advised to return to sublingual buprenorphine by her doctor. At T3, she expressed disappointed with this change, but, by T5, reported feeling positive again and said she was ready to start reducing her sublingual dosage. The other three participants had been taken off LAIB by their service providers because they had not adhered to their respective treatment protocols (two had missed their injection appointments and one had given a positive drug screen). All three expressed frustration at not being given another opportunity to have the medication, including Participant 25 who complained that her service provider was not listening to, or supporting, her:

“I’m no longer on the Buvidal… I had an appointment that week… I thought I was going back on the injection, but no… They still haven’t put me on it yet… saying funding’s run out… I don’t feel like I’m being listened to there or supported.” (Participant 25, female, T5).

### Goal mediators and moderators

#### Barriers to achieving goals

Participants sometimes identified their own poor health as a barrier to making progress in their LAIB treatment. In this regard, several participants stated that they would need to have professional support with their depression and/or anxiety and others described physical health needs that required attention before they could achieve their goals. For example, Participant 26 stated that her mental health would have to improve before she could return to full-time education or employment, whilst Participant 24 described uncontrolled pain that kept her using heroin:

“Well hopefully getting this pain under control, and then I can completely get off… the heroin, go from there… It’s down to the doctors at the moment to get me stabilised on painkillers.” (Participant 24, female, T4)

Other participants complained that lack of support from treatment services was undermining their ability to work towards their goals. These participants maintained that they needed more contact from their key workers; additional counselling; greater access to support groups; and/or increased opportunities to participate in structured activities. Indeed, several participants described feeling ‘let down’ by treatment providers for not offering this wrap-around support. This included Participant 19 who said that nobody from the service where he was receiving his LAIB ever phoned him to see how he was or to offer other help:

“I just feel so let down by it [treatment service], and if I didn’t have the support network [partner and family], I don’t know where… I’d be.” (Participant 19, male, T4)

In addition, some participants discussed situational factors that made it difficult for them to achieve their goals. Alongside homeless and unstable housing, these included having insufficient support from family and friends; finding it difficult to avoid people who were using drugs; lack of daily routines and/or meaningful activity; the closure of facilities because of COVID-19; and needing to feel more certain about the future. At T4, for example, Participant 23 stated that she wanted to complete her college course and get the next stage of her life in place before she could think about reducing her LAIB:

“Finish my studies and hopefully get something else in line… Maybe after that I’d start to think about reducing… Once I’ve got a sense of direction about what I’m doing.” (Participant 23, female, T4)

#### Enablers to achieving goals

Despite these barriers, participants also described factors that enabled them to make progress in their treatment and move towards their goals. These included partners or family members who provided practical and emotional assistance; interests, hobbies, or college work that gave routine and structure to their days; access to mutual aid meetings where they could meet with others to share experiences; and paid employment which provided income to buy food and material possessions. Many participants also emphasised that their LAIB treatment was itself directly responsible for them beginning to achieve their goals, with the medication sometimes acting as a catalyst to other positive life changes:

“The Buvidal definitely boosts my mood… I’ve got money in my pocket all the time… and that’s unheard of… Sometimes I’d go three, four days without eating, no food in the cupboard. We’ve got food in the cupboard, food in the fridge, food in the freezer… I wake up feeling great… I have breakfast. I never used to have breakfast… The next step… is [to] get back into work.” (Participant 11, male, T3)

## Discussion

Our analyses of the goals patients wanted to achieve from LAIB replicate problems evident in earlier drug treatment literature [39, 40]. Specifically, participants often stated that they wanted to be ‘abstinent’ or ‘drug free’ without clarifying what exactly they meant by this. Mostly, they anticipated receiving LAIB in the short-to-medium term but planned to come off the medication at some point in the future. Although they seldom used the term ‘recovery’ to describe their treatment goals, nearly all articulated objectives (improved relationships, better physical and mental health, stable housing, paid employment etc.) that are consistent with the concept of recovery as is now widely documented in the international literature [15, 16, 41–43]. Those initiating LAIB may therefore not have engaged directly with the language of recovery, but current understandings of recovery seemed to capture their treatment goals.

The longitudinal analyses undertaken revealed that participants tended to identify consistent, albeit sometimes evolving, goals. Additionally, several participants extended the timeframe for beginning to reduce their LAIB at their later interviews. This may have occurred because we did not ask participants to distinguish between their ‘hopes’ and ‘expectations’. Whilst hope refers to a patient’s most ‘desired’ (preference-driven) outcome, expectation describes their most ‘likely’ (probability-driven) outcome [44]. When information confirming or disconfirming hope is in the distant future, people are generally better able to maintain hope. Conversely, when information about the likelihood of achieving outcomes is proximal, people often abandon overly optimistic beliefs and revert to expectation [44, 45]. Our findings may thus reflect the fact that participants ‘hoped’ for reduction and abstinence in the longer-term but did not expect these outcomes in the short-term.

Generally, participants recognised that achieving their treatment goals would take time, not least as they also often had other complex life problems to address. Three participants who had been taken off LAIB by their service providers because of non-adherence to treatment protocols wanted to return to the medication. This suggests that more than one treatment episode may be necessary. The range of (often material and structural) problems reported by participants supports the argument that people cannot ‘recover’ from an addictive behaviour through individual actions or a change in mindset alone [19]. Rather, patients need systems and structures (including supportive relationships, meaningful activity, income, and physical possessions) to enable behaviour change. In the recovery literature, these resources have been referred to as recovery capital [46, 47]. According to Cloud and Granfield [47], and reflected in our participants’ accounts, people who have access to recovery capital seem better placed to address problems related to substance use.

Overall, participants appeared to understand the various resources they required to achieve their treatment goals. Their insights in this regard, and in relation to the factors impeding their treatment progress, highlight good opportunities for person-centred care [28]. This is because those who are informed and who can articulate their needs have greater ability to participate in discussions and decisions about their own treatment. Some people may, of course, want or require additional information about LAIB to improve their decision-making [34, 48]. Meanwhile, others may not feel ready or able to make treatment choices and may prefer their doctors to make treatment decisions for them [34]. Consequently, it is important to promote personalised care amongst patients who want to be more involved without pressurising those who do not. For some, participation may thus be limited to checking that they are feeling comfortable with the direction of treatment. For others, it may involve regular planning meetings to discuss and modify care plans or to understand why they are finding it difficult to adhere to their treatment protocol and revising this if possible.

In terms of more general recommendations for policy and practice, participants’ limited consideration of the meaning of ‘abstinence’ and infrequent use of the word ‘recovery’ together indicate a need for wider debate regarding the goals those initiating LAIB are seeking and the diverse range of positive treatment outcomes LAIB could potentially offer. Policy makers, commissioners, service providers and patients may all benefit from (i) engaging in more in-depth discussions with each other about concepts such as ‘abstinence’ and ‘recovery’, (ii) recognising that these will mean different things to different people, and (iii) acknowledging that an individual’s goals in relation to their substance use and life more broadly may change over time [15, 16, 41–43]. Furthermore, patients will often want and need on-going assistance from treatment services and other professionals, including with a wide range of life issues. It is therefore essential that those providing LAIB continue to offer regular contact and additional forms of non-medical support so that patients are given the best opportunity to succeed and do not feel abandoned. Inevitably, there will be constraints (regulatory, financial, and organisational etc.) on what treatment services can offer [26, 31–33] and patients may sometimes need to accept that they cannot always be given the help they prefer. Despite this, policies of recovery and person-centred care should mandate professionals to ascertain what patients themselves want to achieve, to work with them holistically and flexibly, to involve them in treatment decision-making, and to tailor support to their individual needs and preferences whenever possible [29–32].

### Limitations

As with any study, our findings have limitations. Data were generated when LAIB was relatively new in the UK and availability was restricted. People receiving the treatment at the time (and thus those interviewed) may have been selected by treatment providers because they seemed most likely to benefit. As a result, the views and experiences of those interviewed may differ from those initiating the treatment in other contexts. In addition, we did not ask participants to distinguish between their ‘hopes’, ‘expectations’, ‘goals’, and ‘plans’. Nor did we specifically ask them about ‘recovery’ or ‘person-centred’ care. Instead, we invited people to tell us what they hoped and expected to achieve from LAIB and followed up with general prompts and probes. As an early exploration of treatment goals, this seemed an appropriate approach. However, future research might benefit from being more precise in its use of terms and from investigating patients’ goals through a more explicitly structural lens where political, economic, legal, and cultural relations are discussed more directly [19]. Finally, we have not presented data on whether participants achieved their treatment goals. In due course, our analyses of twelve-month (T6) interview data will enable us to present one-year outcomes.

## Conclusions

LAIB is an emerging new medication for opioid use disorder, and it is important to ascertain what people want to achieve from the treatment. Participants often articulated a desire to be abstinent and identified broader life goals consistent with the concept of ‘recovery’ [15, 16, 41–43]. Policies of ‘recovery’ and ‘person-centred care’ have gained prominence across the addiction field and provide the backdrop to current UK drug treatment. However, they have been criticised for placing too much onus on patients and people who use services to take better care of themselves and to change their own lives [19, 20, 22, 23, 26, 31, 32]. Our research found that people initiating LAIB had good insights into their own treatment needs and views on their personal care; yet they did not report feeling responsible when they did not achieve their treatment goals quickly. Instead, they delayed their plans to reduce medication, identified additional assistance that would help them, and expressed frustration at services and systems for failing them. Rather than making patients feel responsible for improving their own lives, it seems possible that policies relating to recovery and person-centred care may, in fact, be empowering patients to expect, and appreciate that they are deserving of, a greater range of support.

## Data Availability

The dataset generated and analysed during the current study is not publicly available due to small sample size, sensitive data, and potential identification of organisations and individuals contra confidentiality agreements. Please contact the corresponding author for further information.

## List of abbreviations

LAIB: Long-acting injectable buprenorphine
T: Timepoint
UK: United Kingdom
